# Novel Evidence That Attributing Affectively Salient Signal to Random Noise Is Associated with Psychosis

**DOI:** 10.1371/journal.pone.0102520

**Published:** 2014-07-14

**Authors:** Ana Catalan, Claudia J. P. Simons, Sonia Bustamante, Marjan Drukker, Aranzazu Madrazo, Maider Gonzalez de Artaza, Iñigo Gorostiza, Jim van Os, Miguel A. Gonzalez-Torres

**Affiliations:** 1 Department of Neuroscience, University of the Basque Country, Basque Country, Spain; 2 Department of Psychiatry, Basurto University Hospital, Bilbao, Spain; 3 Department of Psychiatry and Psychology, South Limburg Mental Health Research and Teaching Network, EURON, Maastricht University Medical Centre, Maastricht, The Netherlands; 4 GGzE, Eindhoven, The Netherlands; 5 Research Unit REDISSEC, Basurto University Hospital, Bilbao, Spain; 6 King’s College London, King’s Health Partners, Department of Psychosis Studies, Institute of Psychiatry, London, United Kingdom; University of Groningen, Netherlands

## Abstract

We wished to replicate evidence that an experimental paradigm of speech illusions is associated with psychotic experiences. Fifty-four patients with a first episode of psychosis (FEP) and 150 healthy subjects were examined in an experimental paradigm assessing the presence of speech illusion in neutral white noise. Socio-demographic, cognitive function and family history data were collected. The Positive and Negative Syndrome Scale (PANSS) was administered in the patient group and the Structured Interview for Schizotypy-Revised (SIS-R), and the Community Assessment of Psychic Experiences (CAPE) in the control group. Patients had a much higher rate of speech illusions (33.3% versus 8.7%, OR_adjusted_: 5.1, 95% CI: 2.3–11.5), which was only partly explained by differences in IQ (OR_adjusted_: 3.4, 95% CI: 1.4–8.3). Differences were particularly marked for signals in random noise that were perceived as affectively salient (OR_adjusted_: 9.7, 95% CI: 1.8–53.9). Speech illusion tended to be associated with positive symptoms in patients (OR_adjusted_: 3.3, 95% CI: 0.9–11.6), particularly affectively salient illusions (OR_adjusted_: 8.3, 95% CI: 0.7–100.3). In controls, speech illusions were not associated with positive schizotypy (OR_adjusted_: 1.1, 95% CI: 0.3–3.4) or self-reported psychotic experiences (OR_adjusted_: 1.4, 95% CI: 0.4–4.6). Experimental paradigms indexing the tendency to detect affectively salient signals in noise may be used to identify liability to psychosis.

## Introduction

Despite decades of research, the mechanism of hallucinations remains unclear [1]. Recent theories suggest that hallucinations are due to a dysregulation in top-down processing, when a higher priority is assigned to the top-down process in the final perception [2], [3]. Several studies have reported that hallucinations in schizophrenia are due to difficulties in distinguishing between an internally generated event and a real event [4], [5], but it has been argued that reality-monitoring represents a consequence rather than a cause of the disorder [3]. A recent study has found that the tendency to detect affectively salient speech illusions in random noise was more prevalent in patients with a psychotic disorder, and in addition was associated with higher levels of positive schizotypy in healthy subjects, independent of measures of neurocognition. These results therefore suggest that white noise speech illusion reflects individual differences in the risk of developing psychotic symptoms [6].

Several approaches toward experimental assessment of speech illusions have been reported. Hoffman [7] described an experimental design measuring individual differences in picking up speech illusions from multi-speaker babble and showed that illusions predicted transitions to a schizophrenia-spectrum disorder in individuals with prodromal psychosis. Another approach involves the detection of speech stimuli embedded in noise in the context of a speech recognition paradigm. A further variant of the ‘false-positive meaning’ approach is the experiment in which pure white noise is presented and individuals indicate whether or not they perceive spoken words [6], [8].

A further variant includes experimental induction of salience in the illusion of remembering a stimulus not presented before. Semantic expectations play a role in priming hallucinatory perceptual experiences. Short sentences were presented in which the penultimate word was masked with white noise. The subjects were asked to identify the target word. The authors observed increased top-down influence on perception of auditory verbal stimuli in hallucination-prone controls [8].

In the current investigation, an extension of the ‘false-positive meaning’ approach was used, as described in a previous paper introducing the ‘white noise test’ [6]. The aim of the current study was to replicate these results [6], describing the prevalence of speech illusions in patients with a first episode of psychosis (FEP) and controls, particularly of speech illusions in which additional ‘emotional meaning’ was detected. In addition, we wished to assess the association between speech illusions and psychosis in patients (using measures of psychotic symptoms) and controls (using measures of schizotypy), independent of cognitive performance.

## Materials and Methods

### Ethics statements

The local ethics committee (Ethics Committee of Clinical Research of Basurto University Hospital) approved the study design and the patients provided written informed consent. We obtained written consent from all patients and in the case of minors from their guardians as well.

### Sample

Data were collected from a convenience sample of patients with a FEP admitted consecutively to the inpatient unit of Basurto University Hospital from January 2009 to September 2012. Controls were recruited from the general population in the same catchment area of the patients, through public announcements. They were similar in age and sex to the patients, and did not have first-degree family members with a psychotic disorder. Patients were evaluated when the psychiatrist in charge considered that they were stable enough to be interviewed. Inclusion criteria were the following (for both groups): between 17 and 65 years of age, sufficient mastery of the Spanish language, IQ>70, and for patients: exposure to antipsychotic medication <1 year. In patients, the psychotic episode fulfilled criteria of the DSM-IV-TR for affective or non-affective psychotic disorder. Exclusion criteria were: psychotic episode was the consequence of abuse of drugs or somatic disorder, or not willing to participate.

Interviewers were graduate psychologists and psychiatrists with experience in the use of specific instruments [6].

Sociodemographic variables including age, sex, onset of psychosis, duration of untreated psychosis, work, marital status and area of residence were collected. For the patients, the PANSS scale (yielding scales of positive, negative, general and global symptoms) was used to assess psychopathology. In the analyses, the PANSS positive symptom score was reduced to two groups using the median split of the scale. The Operational Criteria Checklist for Psychosis (OCCPI) was completed, based on clinical scales and relevant data in the medical history, and used to establish the diagnosis of the patients using the associated OPCRIT computer programme.

The main variable of analysis was speech illusions and the attribution of emotional meaning, as assessed in the white noise task. Associations between speech illusions and psychopathology were assessed using the Positive and Negative Symptom Scale (patients) [9], the Structured Interview for Schizotypy-Revised (controls) [10] and the Community Assessment of Psychic Experiences (controls) [11].

### Instruments

#### White noise

Subjects wore earphones and were presented 1 of 3 different types of stimuli: (1) white noise only, (2) white noise + clearly audible neutral speech, and (3) white noise + barely audible neutral speech. Stimuli 2 and 3 were not separate conditions; the intermixing of white noise stimuli with audible speech was presented in order to create a higher level of expectation, thus occasioning higher levels of top-down processing. Participants were presented 25 fragments of each, in random order, and were asked to respond to each by pressing 1 of 5 buttons hereafter referred to as 1: positive speech illusion (endorsed hearing positive voice), 2: negative speech illusion (endorsed hearing negative voice), 3: neutral speech illusion (endorsed hearing neutral voice), 4: no speech heard, and 5: heard speech but uncertain whether voice was positive, negative or neutral; this latter option was included in order to make the ratings of 1–3 more conservative. Each fragment of noise lasted approximately 5 seconds. The recordings were delivered using stimulation software E-prime 1.1 (Psychology Software Tools, Pittsburgh, Pennsylvania), and stimuli were reproduced in random order. The length of the task was approximately 15 minutes. The rate of hearing a voice in the white noise–only condition (25 trials) was the variable of interest in the analyses. A dichotomous variable was created (speech illusion present versus not present) in which a speech illusion was considered a positive result. With the objective of making the task more specific (excluding possible false positive results) a restrictive criterion was set excluding, from the group of any speech illusions, those subjects who only heard an illusion once.

#### SIS R

Structured Interview for Schizotypy–Revised. The Structured Interview for Schizotypy–Revised was used to determine a broad range of schizotypal symptoms and signs. Items can be scored on a 4-point scale from absent (0) to severe (3). Positive schizotypy covers the symptoms referential thinking (2 items), magical ideation, illusions, psychotic symptoms, and suspiciousness (6 items). Negative schizotypy covers the symptoms of social isolation, introversion, restricted affect, and poverty of speech (4 items). Mean schizotypy scores for these dimensions were calculated, resulting in a positive schizotypy and a negative schizotypy score. In the analyses, SIS-R positive symptom score was used, divided by its median value, creating median groups.

#### CAPE

Community Assessment of Psychic Experiences [12] was used to assess the lifetime prevalence of positive and negative and depressive symptoms. This self-reporting scale measures positive and negative and depressive symptoms on both a frequency scale (0 = never to 4 = nearly always) and a distress scale (1 = not distressed to 4 = very distressed). In the analyses, cape positive symptom score was used, divided by its median value, creating median groups.

#### IQ

The short form of the Wechsler Adult Intelligence Scale – III [13] was assessed for an indication of intellectual functioning (IQ), and included the following tests: ‘Block Design’, ‘Digit Symbol’, ‘Arithmetic’ and ‘Information’.

### Analyses

Socio-demographic differences between groups were assessed. A Kolmogorov-Smirnov test was used to test for normality of variables. Student’s t-test was used to examine differences in continuous variables, and Mann-Whitney’s U-test was used for non-normally distributed variables. In the case of categorical variables, chi square tests, and Fisheŕs exact test when necessary, were performed.

Group differences in percentage of speech illusions and affectively salient illusions were assessed with Fisheŕs exact test. As white noise speech illusion scores for positive, negative, and neutral voices were highly skewed, the 3 outcomes were analyzed as dichotomous variables, conform previous work [6]. A variable “any speech illusion” was constructed denoting the presence of at least two instances of any positive, negative, or neutral voice perceived in white noise.

In order to assess whether the white noise task was sensitive particularly to affectively salient speech illusions rather than neutral speech illusions, a composite variable was constructed reflecting any positive or negative speech illusions.

Case-control status was the binary response variable and (affectively salient) white noise speech illusion the binary exposure variable in logistic regression models, all adjusted for age and sex. In the group comparison of affectively salient speech illusions, non-affectively salient speech illusions were excluded from the analysis. In order to test whether case-control differences were reducible to cognitive alterations, models were additionally adjusted for WAIS-IQ score. Adjusted ORs were obtained by adding the confounders to the logistic regression model.

In order to assess, in the patient group, whether speech illusions were associated with the binary PANSS positive symptom variable, logistic regression analyses were run with speech illusions as the dependent variable the binary PANSS-positive symptom variable as independent variable.

In order to assess, in the control group, the association between white noise speech illusion on the one hand, and binary schizotypy and binary CAPE positive symptoms on the other, multilevel logistic regression models of “any speech illusion” were run, adjusted for age and sex.

The statistical analyses were carried out using the stata software programme, version 11 [14].

## Results

### Sample

Patients and control subjects were approximately similar in age, sex and years of education while differing in their marital status, socio-economic level, residence and intelligence quotient (IQ) (Table 1). Diagnoses in the group of patients were the following: schizophrenia or schizophreniform disorder (n = 32), affective psychoses (n = 14), brief psychotic episode (n = 2) and delusional disorder (n = 6). Mean age of illness onset was 31.6 (SD = 11.5). All patients were taking antipsychotic treatment at the time of the assessment. The mean PANSS global score was 78.6 (SD = 18.9), and subscores were: general 39.4 (SD = 11.2), negative 11.5 (SD = 7.7) and positive 28.2 (SD = 9.1) (Table 2).

**Table 1:** Socio-demographic and cognition variables (N [%] or average [SD]).

		*Patients (N = 54)*	*Controls (N = 150)*
Sex	Male	37 (68.5%)	87 (58.0%)
	Female	17 (31.5%)	63 (42.0%)
Age (years)		34.7 (12.6)	33 (11.3)
Years of education		12.2 (3)	14.8 (2.4)
Socio-economic level	High middle class	9 (16.7%)	28 (18.7%)
	Middle class	32 (59.2%)	109 (72.7%)
	Low middle class	13 (24.1%)	13 (8.6%)
Marital status	Single	37 (68.5%)	83 (55.3%)
	Married/Partner	11 (20.4%)	64 (42.7%)
	Divorced	4 (7.4%)	3 (2%)
	Widower	2 (3.7%)	0
Work status	Inactive	27 (50%)	20 (13.3%)
	Active	25 (46.4%)	85 (56.7%)
	Student	1 (1.8%)	40 (26.7%)
	Others	1 (1.8%)	2 (1.3%)
Residence	Parents	31 (57.4%)	72 (48%)
	Partner/Children	14 (26%)	68 (45.3%)
	Alone	9 (16.6%)	10 (6.7%)
WAIS-IQ		94.1 (16.6)	110.2 (14.7)

WAIS IQ, intelligent quotient in Wechsler adult scale.

**Table 2:** Clinical sample description.

		FEP			CONTROLS			
		*x¯* (SD)	[min–max]	median		*x¯ *(SD)	[min–max]	median
PANSS	Positive	28.2 (9.1)	[9–49]	28	CAPE Positive*	4 (2.7)	[0–15]	4
	Negative	11.5 (7.7)	[7–36]	8	SIS-R Positive**	1.5 (1.7)	[0–7]	1
	General	39.4 (11.2)	[20–81]	38.5				
	Global	78.6 (18.9)	[47–142]	75				
WAIS III		94.1 (16.7)	[70–131]	95.5		110.2 (14.7)	[73–149]	111

*x¯* = mean, SD = standard deviation.

* = CAPE Positive scale frequency score.

** = SIS-R positive scale score.

### Speech illusions

Patients had a much higher rate of speech illusions than controls (33.3% versus 8.7%, OR_adjusted_: 5.1, 95% CI: 2.3–11.5, p<0.0001), which was only partly explained by differences in IQ (OR_adjusted_: 3.4, 95% CI: 1.4–8.3, p = 0.0003).

Speech illusions in the patient group were associated, albeit just short of statistical significance, with PANSS positive symptom group (OR_adjusted_: 3.3, 95% CI: 0.9–11.6; p = 0.068). This association was not apparent for general or negative PANSS symptoms (OR_adjusted_ general: 0.6, 95% CI: 0.2–2.1; OR_adjusted_ negative: 2.0, 95% CI: 0.6–7.0).

### Affectively salient speech illusions

Patients had a much higher rate of affectively salient speech illusions than controls (9.3% versus 1.3%, OR_adjusted_: 9.7, 95% CI: 1.8–53.9, p<0.01), which was only partly explained by differences in IQ (OR_adjusted_: 6.5, 95% CI: 0.98–43.8, p = 0.053).

Affectively salient speech illusions in the patient group were associated, albeit short of statistical significance, with PANSS positive symptom group (OR_adjusted_: 8.3, 95% CI: 0.7–100.3, p = 0.26).

### Schizotypy and speech illusions in controls

Speech illusions in controls were associated with neither the SIS-R positive scale (OR_adjusted_: 1.1, 95% CI: 0.3–3.4, p = 0.9) nor the CAPE positive scale (OR_adjusted_: 1.4, 95% CI: 0.4–4.6, p = 0.84).

## Discussion

Patients with FEP demonstrated higher rates of speech illusions than the group of control subjects, particularly speech illusions perceived as affectively salient. Differences were only in part reducible to differences in cognition. These results agree with those found by Galdos et al. [6]. In patients, speech illusions were associated with positive symptomatology (PANSS scale), which was not the case in controls, contrary to the report by Galdos and colleagues [6].

The most commonly reported difference between healthy and clinical voice hearers (AVH) is the emotional valence of the voice [15]–[17], a negative emotional appraisal of the voice having a predictive value of 88% for the presence of a psychotic disorder [18]. The formation of delusions may be due to aberrant salience, or attributed importance, to speech illusions [19]. We cannot determine to what degree the mechanisms underpinning speech illusions in healthy participants are the same as those demonstrated in patients. Speech illusions were only associated with psychotic symptoms when they required care. Therefore, non-clinical speech illusions in and of themselves cannot be taken to indicate risk of psychotic disorder. Additional clues to the developmental trajectories which differentiate clinical from healthy AVH can be derived from consideration of the phenomenology, cognitive mechanisms, and emotional regulation differences between the two populations.

Absence of association between speech illusions and measures of psychosis in the healthy participants (n = 150) requires follow-up. Interestingly, Galdos and colleagues [6] did report a positive association in a larger and younger group of controls. The SIS-R and CAPE scales are designed to measure prevalence of positive experiences in general population, with average specificity and sensibility that require relatively large samples in order to detect small effects. A greater number of controls, and of younger age, may be required to demonstrate the hypothesized association [10], [11], [20]. However, the power to detect differences might be improved by selecting participants with high and low schizotypy (positive symptoms), e.g. those in the 4th quartile vs 2nd quartile, rather than using the median split, even if that results in smaller samples. Future research may apply such strategies a priori in order to improve the sensitivity of the analyses.

The inclusion of patients with FEP can be considered a strength, given that they have less exposure to medication, possibly interfering with neuropsychological testing, and case-control differences are unlikely to be the result of chronicity rather than illness onset. FEP patients also have a lower prevalence of positive symptoms that are resistant to treatment. In addition, as the sample was drawn from a defined catchment area population, patients can be considered representative. In conclusion, the white noise task is easy to administer, differentiates between patients and controls, and is specifically associated with positive symptoms in patients. Whether or not it indexes psychosis proneness in healthy participants remains uncertain. More studies are required in order to understand its predictive value in the general population.
